# Assessing the potential of Other Effective area-based Conservation Measures (OECMs) for contributing to conservation targets: A global scoping review protocol

**DOI:** 10.12688/openreseurope.16116.3

**Published:** 2024-01-10

**Authors:** Dimitra Petza, Eva Amorim, Emma Ben Lamine, Francesco Colloca, Esther Dominguez Crisóstomo, Erika Fabbrizzi, Simonetta Fraschetti, Ibon Galparsoro, Sylvaine Giakoumi, Maren Kruse, Vanessa Stelzenmüller, Stelios Katsanevakis

**Affiliations:** 1 Department of Marine Sciences, University of the Aegean, Mytilene, Lesvos, Greece; 2 International Estuarine & Coastal Specialists (IECS) Ltd, Leven, UK; 3 Centre National de la Recherche Scientifique (CNRS), Université Côte d’Azur, Nice, France; 4 Integrative Marine Ecology Department, Stazione Zoologica Anton Dohrn, Rome, Italy; 5 Department of Biology, University of Algarve, Faro, Portugal; 6 Department of Biology, University of Naples Federico II, Naples, Italy; 7 Marine Research Division, AZTI, Pasaia, Spain; 8 Sicily Marine Centre, Stazione Zoologica Anton Dohrn, Palermo, Italy; 9 Thünen Institute of Sea Fisheries, Bremerhaven, Germany

**Keywords:** Other Effective area-based Conservation Measures, conservation targets, scoping review, JBI methodology, PRISMA statement, Kunming-Montreal Global Biodiversity Framework, biodiversity conservation, EU Biodiversity Strategy

## Abstract

This scoping review (ScR) protocol aims to establish the methodological approach for identifying and mapping the evidence regarding the actual contribution of Other Effective area-based Conservation Measures (OECMs) to spatial conservation targets. Emphasis will be placed on examining the research conducted, including the methodologies applied. OECMs, introduced by the Convention on Biological Diversity (CBD) in 2010, refer to areas outside of protected areas, such as fisheries restricted areas, archaeological sites, and military areas, that effectively conserve biodiversity in-situ over the long term. OECMs are recognized rather than designated. Many countries currently endeavor to identify, recognize and report OECMs to the CBD for formal acceptance to support the implementation of spatial conservation targets. Studies that assess the contribution of OECMs to spatial conservation targets will be considered. Potential OECMs with primary, secondary or ancillary conservation objectives established by all sectors in the terrestrial, freshwater and marine realm worldwide will be considered. Peer-reviewed and grey literature will be considered without imposing limitations based on publication year, stage, subject area and source type. Both experimental and observational studies in English, French, German, Greek, Italian, and Spanish will be reviewed. The ScR will follow the Joanna Briggs Institute (JBI) methodology. The protocol will be guided by the PRISMA-ScR (Preferred Reporting Items for Systematic Reviews and Meta-Analyses) extension for scoping reviews. The search will encompass bibliographic databases such as Scopus, Web of Science and Google Scholar. Grey literature sources will include databases, pre-print archives and organizational websites. The Covidence platform will be utilized for data management and extraction.

## Introduction

Other Effective area-based Conservation Measures (OECMs) were introduced in 2010 by the Convention on Biological Diversity (CBD) as areas that achieve long-term and effective *in-situ* biodiversity conservation outside of protected areas ( CBD, 2010). Consequently, OECMs represent a novel conservation approach where conservation outcomes are incidental to existing spatial management practices. In other words, OECMs are identified and recognized rather than specifically designated. The definition, guiding principles, common characteristics and criteria for identifying OECMs were agreed upon by CBD parties in 2018 ( CBD, 2018). According to CBD Decision 14/8 ( CBD, 2018), key criteria for an area to be identified as an OECM include geographic definition, governance and management, achieving positive and sustained long-term outcomes for biodiversity conservation, including associated ecosystem functions, services and locally relevant values such as cultural, spiritual, and socioeconomic aspects where applicable. Subsequently, additional guidance has been developed by the International Union for the Conservation of Nature (IUCN), the Food and Agriculture Organisation (FAO) and other global organizations to facilitate the identification, recognition and reporting of OECMs ^1^ ( FAO, 2019; FAO, 2022; Garcia *et al.*, 2021; ICES, 2021; IUCN-WCPA, 2019) to contribute to the attainment of Target 14.5 of the 2030 Agenda for Sustainable Development of the United Nations ( UN, 2015) and Action Target 3 of the Kunming-Montreal Global Biodiversity Framework ( CBD, 2010; CBD, 2022). The latter emphasizes the need to conserve at least 30% of terrestrial and marine areas globally by 2030 through ecologically representative, effectively and equitably managed, and well-connected networks of protected areas and OECMs ( CBD, 2022).

In recent years there has been increasing research and policy interest in OECMs and numerous countries worldwide have made significant efforts to identify and recognize OECMs to support the implementation of spatially-explicit conservation targets. According to the most recent update of the World Database on Protected Areas (May 2023; WDPA, 2023), 671 OECMs have been recognized by only nine countries worldwide (none in Europe).

This protocol aims to establish the methodological approach for a Scoping Review (ScR) with the following objectives:

- identify and map the available evidence on assessing the potential of OECMs to contribute to spatial conservation targets,
- examine the methodologies employed in research on assessing potential OECMs,
- identify the actual spatial contribution of potential OECMs to conservation targets,
- provide insights into the evidence-based knowledge about OECMs and information on how potential OECMs contribute to the spatial targets set by CBD.

## Review question

The overall research question that will guide the ScR is: What is the current knowledge regarding the contribution of OECMs to biodiversity conservation targets? The ScR will aim to address the following sub-questions:

1. What is the geographical distribution of studies that have assessed potential OECMs and their contribution to biodiversity conservation?
2. What are the characteristics of the potential OECMs studied in terms of governance type, sector, realm, conservation objectives, and rationale?
3. What methodologies have been employed to assess the potential of OECMs in contributing to biodiversity conservation?
4. What is the spatial contribution (percentage of area covered) of the potential OECMs?
5. What are the main outcomes of the studies that have assessed potential OECMs regarding key findings, effectiveness of potential OECMs, gaps of knowledge and policy recommendations?

## Inclusion/exclusion criteria

The inclusion criteria of the ScR, which serve as the basis for determining the sources to be considered for inclusion in the review, will be developed in accordance with the "Participants, Concept and Context (PCC)" mnemonic ( Table 1).

**Table 1.** Inclusion and exclusion criteria for the Scoping Review in correspondence with the "Participants, Concept and Context, PCC" mnemonic and evidence types and sources.

	Inclusion criteria	Exclusion criteria
**P articipants** ***Potential other effective*** *** area-based conservation*** *** measures (OECMs)***	Potential OECMs governed under a range of governance types i.e., by governments (at various levels), private individuals, organizations or companies, indigenous peoples and local communities and shared governance (i.e., governance by various rights holders and stakeholders together). Potential OECMs established by all sectors (e.g., transport, offshore energy, fisheries, aquaculture, maritime, tourism, defence, archaeological heritages, etc.). Potential OECMs with primary, secondary or ancillary conservation objectives.	---
**C oncept** ***Assessing potential OECMs***	All studies that assess potential OECMs. All types of methodologies and metrics applied to assess the effectiveness of potential OECMs to deliver biodiversity conservation outcomes and contribute to spatial conservation targets.	---
**C ontext** ***Global terrestrial,*** *** freshwater and marine*** *** realm***	Studies in: - terrestrial, freshwater and marine realms, - globally	---
**EVIDENCE TYPES &** ** SOURCES**	- peer-review literature - grey literature - all years of publication - all publication stages, subject areas, and source types - experimental and observational studies - studies published in languages competent to the researchers’ team (e.g., English, French, German, Greek, Italian, Spanish, etc.)	Evidence synthesis such as systematic, scoping, rapid, and narrative reviews

### Participants

The ScR will consider potential ΟECMs, established by any sector, such as transport, offshore energy, fisheries, aquaculture, maritime, tourism, defense and archaeological heritages. These potential OECMs may have primary, secondary or ancillary conservation objectives and can be governed by different entities, including governments (at various levels), private individuals, organizations or companies, indigenous peoples and/or local communities, as well as shared governance involving multiple rights holders and stakeholders.

### Concept

The ScR will focus on the assessment of potential OECMs and how their contribution to spatial conservation targets has been addressed in the existing scientific literature. All studies that assess potential OECMs, along with the various methodologies and metrics applied to evaluate their effectiveness in delivering biodiversity conservation outcomes and contributing to spatial conservation targets will be reviewed.

### Context

The ScR will consider studies conducted in the terrestrial, freshwater, and marine realms worldwide.

### Types of sources

This ScR will encompass both scientific (e.g., articles, book chapters, letters, editorials, books, data papers) and grey literature (e.g., non-published academic research, theses, policy papers, organizational papers and reports, conference abstracts and papers). Scientific literature will be sourced from online databases and grey literature from pre-print archives, organizational websites, and web-based search engines, and suggestions from topic experts. There will be no restrictions on publication year, publication stage (final or in press), subject area, or source type. All document types will be considered, except for evidence synthesis such as systematic, scoping, rapid, and narrative reviews. To align with language competence of the authors, only studies written in English, French, German, Greek, Italian, and Spanish will be included in the ScR.

## Methodology

The proposed ScR will follow the methodology outlined by Arksey and O'Malley (2005), as further developed by Levac *et al.* (2010) and the Joanna Briggs Institute (JBI) methodology ( Peters *et al.,* 2020). The ScR encompass the following nine stages, as recommended by the JBI methodology: 1. Defining and aligning the objectives and questions; 2. Developing and aligning the inclusion criteria with the objectives and questions; 3. Describing the planned approach for evidence searching, selection, data extraction and presentation of the evidence; 4. Conducting the evidence search; 5. Selecting the relevant evidence; 6. Extracting the evidence; 7. Analyzing the evidence; 8. Presenting the results; 9. Summarizing the evidence, drawing conclusions and identifying any implications of the findings ( Peters *et al.,* 2020).

The ScR protocol and final review paper will adhere to the Preferred Reporting for Systematic Reviews and Meta-Analyses extension for scoping reviews (PRISMA-ScR) developed by Tricco *et al.* (2018). The SUMARI Protocol Template for Scoping Reviews in Word format ( https://sumari.jbi.global/) was used to guide the development of this ScR protocol.

### Search strategy

The bibliographic search will be conducted in three databases/ platforms, namely: (a) Scopus, (b) Web of Science – Core Collection, and (c) Google Scholar. A combination of keywords will be used in the search, adapted to meet the specific search specifications of each database. The search will be conducted within the title, abstract and keywords of the documents ( Table 2). For the Scopus and Web of Science databases, all documents retrieved from the search will be considered for eligibility. In the case of the web-based search using the Google Scholar database, only the first 100 hits will be considered ( Haddaway *et al.*, 2015). Eligible documents will also be sought in other sources such as organizational libraries and websites, preprint archives, documents repositories, reference lists of the included documents from the databases search and documents suggested by topic experts and stakeholders.

**Table 2.** Details of Scoping Review search strategy per database, i.e., name of the database, date of search, search query, and results (as the number of documents returned by the search).

Database 1:	**Scopus**
Date of search:	March 19, 2023
Query:	TITLE-ABS-KEY ("other effective area-based conservation measure*" OR "other effective area based conservation measure*" OR "other conservation measure*" OR "OECM*" OR "OEABCM*")
Results:	351 documents
Database 2:	**Web of Science – Core Collection**
Date of search:	March 19, 2023
Query:	TS=("other effective area-based conservation measure*" OR "other effective area based conservation measure*" OR "other conservation measure*" OR "OECM*" OR "OEABCM*")
Results 1:	229 documents
Database 3	**Scholar Google**
Date of search:	March 19, 2023
Query	conservation ("other effective area-based conservation measure*" OR "other effective area based conservation measure*" OR "other conservation measure*" OR "OECM*" OR "OEABCM*")
Results:	996 documents (only the first 100 hits were considered)

### Study/source of evidence selection

Following the search, all identified citations will be uploaded to Covidence, a web-based collaboration software platform designed to streamline the production of systematic and other literature reviews. Any duplicate citations will be removed during this stage. The document selection process will be conducted using a team approach, as recommended by Levac *et al.* (2010). Twelve independent reviewers will be involved in the selection process. Two reviewers will initially screen each title and abstract of the identified papers against the predefined inclusion criteria ( Table 1). Papers that meet the inclusion criteria will proceed to the next stage. The full text of the initially selected documents will be carefully assessed by the reviewers against the inclusion criteria. Any sources that do not meet the inclusion criteria will be excluded from the review. Detailed records will be kept of the reasons for excluding specific sources, and this information will be reported in the final ScR paper. In case of any disagreements between the reviewers during any stage of the selection process, a third independent reviewer will be consulted to resolve the conflicts. The results of the search and the document selection process will be reported comprehensively in the final ScR paper. A flow diagram following the Preferred Reporting Items for Systematic Reviews and Meta-analyses extension for scoping review (PRISMA-ScR) guidelines ( Tricco *et al.*, 2018) will be presented to illustrate the search and selection process.

### Data extraction

Data extraction from the documents included in the ScR will be carried out by two independent reviewers using a data extraction tool, i.e., a charting table aligned to the objective and the questions of the ScR (see Extended data ( Petza *et al.,* 2023)). The data extracted will include specific details related to the participants, concept, context, study methods and key findings relevant to the review objective. To ensure consistency and facilitate collaboration and interaction among reviewers, the data extraction tool will be integrated into the Covidence systematic review management software. This software will help maintain consistency in the extraction process, allow for seamless cooperation between the reviewers, and ensure that the extracted data is consistent and aligned with the objectives and questions of the ScR.

### Data analysis and presentation

The evidence synthesized through the ScR will be presented in alignment with the review objective and specific questions at the final review paper. The full set of the raw data that will be collected by this ScR will be available open-access as a supplementary to the final review paper. The data collected will be analyzed by applying descriptive statistics methods. The summarized data will be presented using a combination of graphical and tabular formats, utilizing appropriate software packages and tools (e.g., Miscosoft Excel, Flourish Studio, Datawrapper Plotly etc.). Graphical representations, such as bar charts, line graphs, donut charts, sankey, chord and network diagrams, choropleth maps, word clouds etc., will be used to visually display relevant information and trends identified in the included studies. These visuals can help convey patterns, relationships, and key findings effectively. For example, the number of documents included in the ScR by year of publication will be presented using bar charts. Choropleth maps will be used to present the geographical distribution of the various case studies reviewed. The different types of OECMs will be depicted using word clouds. Sankey diagrams will be constructed to visualize the flow of information between multiple entities (e.g., conservation objective, realm and sector), while network and chord diagrams will be used to depict the connections between the different methodologies applied for the assessment of potential OECMs. In addition to the graphical and tabular presentations, a narrative summary will be included. This summary will provide a coherent and comprehensive description of the findings, explaining how the results align with the review's objective and specific questions. It will offer a synthesis of the key themes, trends, and patterns identified in the included studies.

## Data Availability

No data is associated with this article. Open Science Framework (OSF): Assessing the potential of other effective area-based conservation measures for contributing to conservation targets: a global scoping review protocol – PRISMA-ScR Checklist and Data Extraction Tool. https://doi.org/10.17605/OSF.IO/3WK5H ( Petza *et al.*, 2023). This project contains the following extended data: Data Extraction Tool.pdf (Data extraction tool of the Scoping Review (ScR)) Open Science Framework (OSF): PRISMA-ScR checklist for ‘Assessing the potential of Other Effective area-based Conservation Measures for contributing to conservation targets: A global scoping review protocol’. https://doi.org/10.17605/OSF.IO/3WK5H ( Petza *et al.*, 2023). Data are available under the terms of the Creative Commons Zero “No rights reserved” data waiver (CC0 1.0 Public domain dedication).
