# Supplementary material for: Assessing the potential of Other Effective area-based Conservation Measures (OECMs) for contributing to conservation targets: A global scoping review protocol
Source: Open Res Eur. 2024 Jan 10;3:118. Originally published 2023 Jul 21. [Version 3] doi: 10.12688/openreseurope.16116.3 (PMC10539721; doi:10.12688/openreseurope.16116.3)
Supplement: Peer review history

# Reviewer report: Reviewer response for version 3

**DOI:** 10.21956/openreseurope.18491.r37383

**Published:** 2024-01-15

**Authors:** Siyuan He

**Affiliations:** 1 Institute of Geographic Sciences and Natural Resources Research, Chinese Academy of Sciences Beijing, Beijing, China

I think the authors have made appropriate modifications.

Is the study design appropriate for the research question?

Yes

Is the rationale for, and objectives of, the study clearly described?

Yes

Are sufficient details of the methods provided to allow replication by others?

Partly

Are the datasets clearly presented in a useable and accessible format?

Not applicable

Reviewer Expertise:

My area of expertise is community-based conservation and sustainable livelihoods in protected areas. One concern is the potential contribution of traditional agrosystems to the protected area management.

I confirm that I have read this submission and believe that I have an appropriate level of expertise to confirm that it is of an acceptable scientific standard.

# Reviewer report: Reviewer response for version 2

**DOI:** 10.21956/openreseurope.18290.r36633

**Published:** 2024-01-03

**Authors:** Siyuan He

**Affiliations:** 1 Institute of Geographic Sciences and Natural Resources Research, Chinese Academy of Sciences Beijing, Beijing, China

Since the goal of assessing the potential of OECMs to achieve "conservation targets" is confined to "spatial conservation" in this review protocol, my first and second concerns do not matter anymore. My third comment was replied by deleting question 5 about good practice and failure. It seems the entire review protocol focused more on the "outcome", but some information on factors that deliver these outcomes can be extracted from Asking Question Five differently. As I think exploring the extent of evidence and mapping it is important for a scoping review, I was wondering if there can be such kind of question.

Is the study design appropriate for the research question?

Yes

Is the rationale for, and objectives of, the study clearly described?

Yes

Are sufficient details of the methods provided to allow replication by others?

Partly

Are the datasets clearly presented in a useable and accessible format?

Not applicable

Reviewer Expertise:

My area of expertise is community-based conservation and sustainable livelihoods in protected areas. One concern is the potential contribution of traditional agrosystems to the protected area management.

I confirm that I have read this submission and believe that I have an appropriate level of expertise to confirm that it is of an acceptable scientific standard, however I have significant reservations, as outlined above.

# Response

**Published:** 2024-01-07

**Authors:** Dimitra Petza

**Affiliations:** Department of Marine Sciences, University of the Aegean, Lesvos, Mytilene, Greece

*We would like to thank the reviewer for the insightful comments. Our response is provided in the following text, where we tried to address all comments one by one, revising also the manuscript accordingly.* **Comment 1. Since the goal of assessing the potential of OECMs to achieve "conservation targets" is confined to "spatial conservation" in this review protocol, my first and second concerns do not matter anymore. My third comment was replied by deleting question 5 about good practice and failure. It seems the entire review protocol focused more on the "outcome", but some information on factors that deliver these outcomes can be extracted from Asking Question Five differently. As I think exploring the extent of evidence and mapping it is important for a scoping review, I was wondering if there can be such kind of question.** *Thank you for your valuable feedback. Following your comment regarding research sub-question 5, we have reconsidered its content and rephrased it as follows: 5. What are the main outcomes of the studies that have assessed potential OECMs regarding key findings, effectiveness of potential OECMs, gaps of knowledge and policy recommendations? This sub-question, which aligns with fields 28-32 of the protocol’s data extraction tool (see here: https://osf.io/4grke), has been added to the revised manuscript (see section “Review question”).*

# Reviewer report: Reviewer response for version 1

**DOI:** 10.21956/openreseurope.17399.r34909

**Published:** 2023-09-28

**Authors:** Siyuan He

**Affiliations:** 1 Institute of Geographic Sciences and Natural Resources Research, Chinese Academy of Sciences Beijing, Beijing, China

The authors provided a protocol for a scoping review to assess the actual contribution of Other Effective Area-based Conservation Measures (OECMs) to spatial conservation targets. Although introduced as early as 2010, this novel conservation approach was detailed much later in 2018 and is now gaining increasing attention as the spatial conservation target (the so-called 3030 target) urges new actions for effective conservation management. There is now growing discussion on the possibility of identifying OECMs, as the criteria include multiple aspects from clear governance to various conservation outcomes. Meanwhile, countries such as Canada and Japan, have commenced recognising OECMs and integrating them into the protected area networks. As there is differentiated conservation responsibility among countries due to biodiversity values, socioeconomic conditions, etc., it is necessary to map the contribution of OECMs globally and provide implications for conservation practice, notably in Europe where none OECMs were registered. This proposed protocol is thus helpful to facilitate evidenced-based spatially-explicit conservation practices in future.

 The protocol is generally well-written and easy to follow. The overall question about the contribution of OECMs to biodiversity conservation targets is further divided into five dimensions that should be addressed during the scoping review. The review procedure is comprehensive and robust following the agreed methodology. I would like to comment on a few points for further consideration:

1. As question three focuses on mapping the methodological approaches that assess the potential outcomes of OECMs, I was wondering if the contributions here include not only spatial extension and connectivity but also ecological functions and other relevant values.
2. Following the first comment, it may be more comprehensive to address multiple conservation outcomes besides the percentage of area covered in Question Four.
3. Question 5 addresses “good practices and failures”, however, I am not sure whether we are talking about the good or bad methods to assess potential OECMs or about the efficiency of OECM management to deliver conservation outcomes. In addition, defining “good” or “bad” according only to the authors of literature seems no practical effect. Addressing antecedents to the success or failures may be more useful.
4. I understand that there was no scanning done after the initial presentation of the database (Table 2) based on the search query, but I was wondering if the current Search Strategy, especially the keywords used, can deliver both comprehensive and targeted databases.

Is the study design appropriate for the research question?

Yes

Is the rationale for, and objectives of, the study clearly described?

Yes

Are sufficient details of the methods provided to allow replication by others?

Partly

Are the datasets clearly presented in a useable and accessible format?

Not applicable

Reviewer Expertise:

My area of expertise is community-based conservation and sustainable livelihoods in protected areas. One concern is the potential contribution of traditional agrosystems to the protected area management.

I confirm that I have read this submission and believe that I have an appropriate level of expertise to confirm that it is of an acceptable scientific standard, however I have significant reservations, as outlined above.

# Response

**Published:** 2023-11-30

**Authors:** Dimitra Petza

**Affiliations:** Department of Marine Sciences, University of the Aegean, Lesvos, Mytilene, Greece

*We would like to thank the reviewer for the insightful comments. Our responses are provided in the following text, where we tried to address all comments one by one, revising also the manuscript accordingly.* **Comment 1. As question three focuses on mapping the methodological approaches that assess the potential outcomes of OECMs, I was wondering if the contributions here include not only spatial extension and connectivity but also ecological functions and other relevant values.**

**Comment 2. Following the first comment, it may be more comprehensive to address multiple conservation outcomes besides the percentage of area covered in Question Four.**

*Question 3 is broad and concerns the review of the methodologies that have been employed worldwide to assess the contribution of OECMs to biodiversity conservation (not the assessment of OECMs' potential outcomes, spatial extension or connectivity). This question is further detailed in the “Concept” subsection where it is stated that “All studies that assess potential OECMs, along with the various methodologies and metrics applied to evaluate their effectiveness in delivering biodiversity conservation outcomes and contributing to spatial conservation targets will be reviewed.”. Question 4 deals with the spatial contribution of the potential OECMs assessed by the studies included in the ScR. It will serve as a means to measure the contribution of the reviewed studies towards the attainment of the spatial conservation targets set by the Global Biodiversity Framework. We hope this clarifies your concerns raised by comments 1 and 2.* **Comment 3. Question 5 addresses “good practices and failures”, however, I am not sure whether we are talking about the good or bad methods to assess potential OECMs or about the efficiency of OECM management to deliver conservation outcomes. In addition, defining “good” or “bad” according only to the authors of literature seems no practical effect. Addressing antecedents to the success or failures may be more useful.**

*Review question 5 has been deleted from the revised manuscript, following the reviewer’s No2 second comment.*

**

**Comment 4. I understand that there was no scanning done after the initial presentation of the database (Table 2) based on the search query, but I was wondering if the current Search Strategy, especially the keywords used, can deliver both comprehensive and targeted databases.**

*As the OECMs, which is the participant of this scoping review, is a new term in the literature and thus we expected to have a small number of relevant studies, we preferred to apply a broad-term approach for our search strategy to avoid losing pieces of relevant evidence. In Table 2, we present the search strategy we developed for each one of the three basic databases that we will use to perform the document search (i.e., Scopus, Web of Science Core Collection and Google Scholar), the adjustments of the queries based on the search specifications of each database, and the results of the pilot searches we ran during the development of the ScR protocol. The queries will be further adapted to the rest of the databases/ repositories that will be searched and will be documented in the final review paper.*

# Reviewer report: Reviewer response for version 1

**DOI:** 10.21956/openreseurope.17399.r34908

**Published:** 2023-09-07

**Authors:** Matthew James Grainger

**Affiliations:** 1 Norwegian Institute for Nature Research, Trondheim, Norway

The article is a protocol for a "global scoping review" addressing the primary question - "What is the current knowledge regarding the contribution of 'Other Effective area-based Conservation Measures to biodiversity targets" with 5 sub-questions - namely:

 "1. What is the geographical distribution of studies that have assessed potential OECMs and their contribution to biodiversity conservation?"

 "2. What are the characteristics of the potential OECMs studied in terms of governance type, sector, realm, conservation objectives, and rationale?"

 "3. What methodologies have been employed to assess the potential of OECMs in contributing to biodiversity conservation?"

 "4. What is the spatial contribution (percentage of area covered) by potential OECMs?"

 "5. What are the good practices and failures acknowledged in the literature?"

 In general, this is a well written protocol which appears to be robust and follows current guidance for Systematic Evidence Synthesis in environmental fields (although I recommend referring to the CEE guidelines (systematic map guidelines are closest to a scoping review)). 

 Given that this is a Scoping Review I do not understand how the authors intend to achieve objective 5 - "What are the good practices and failures acknowledged in the literature?". There is no critical appraisal of the studies included proposed and therefore it would be difficult to assess (beyond what the individual study authors suggest in each included paper) what is "good practice" (or "bad practice"). 

 I find the structure of the protocol a bit odd - I would expect to read about the search strategy first and then read about inclusion criteria - but perhaps this is a set template.

 Again there are some subheadings that appear a little redundant which might be due to a template being used - for example "Types of sources" doesn't include the actual databases (but talks about databases) and it is only when you get to the search strategy that this is mentioned. There is considerable scope for streamlining the document. Another example: "Language limitations will be applied during the literature search process to align with language competence of the authors. Consequently, studies published in languages other than English, French, German, Greek, Italian, and Spanish will be excluded from the ScR." Why not: "To align with language competence of the authors only studies written in  English, French, German, Greek, Italian, and Spanish will be included".

 Study/source of evidence selection - if the authors plan to do forward and backward citation chasing then I recommend using citationchaser | Evidence Synthesis Hackathon (eshackathon.org)

 What is the data extraction tool? Do the authors have an example (even if not complete)? I see that the tool is linked in the data availability - but I would like to see this in the protocol explicitly. 

 There are some vague statements in the Data analysis section - for example "The data will be presented using a combination of graphical and tabular formats, utilizing appropriate software packages and tools. Graphical representations, such as charts, graphs, or diagrams, will be used to visually display relevant information and trends identified in the included studies. These visuals can help convey patterns, relationships, and key findings effectively."

 How will data be presented using what tools and to what aims? I know there needs to be some uncertainty here but the authors can provide more specific details here as at the moment the text is generic and uninformative. 

Is the study design appropriate for the research question?

Partly

Is the rationale for, and objectives of, the study clearly described?

Yes

Are sufficient details of the methods provided to allow replication by others?

Partly

Are the datasets clearly presented in a useable and accessible format?

Not applicable

Reviewer Expertise:

Evidence synthesis with a focus on the environment and conservation

I confirm that I have read this submission and believe that I have an appropriate level of expertise to confirm that it is of an acceptable scientific standard, however I have significant reservations, as outlined above.

# Response

**Published:** 2023-11-30

**Authors:** Dimitra Petza

**Affiliations:** Department of Marine Sciences, University of the Aegean, Lesvos, Mytilene, Greece

*We would like to thank the reviewer for the insightful comments. Our responses are provided in the following text, where we tried to address all comments one by one, revising also the manuscript accordingly.* **Comment 1. In general, this is a well-written protocol which appears to be robust and follows current guidance for Systematic Evidence Synthesis in environmental fields (although I recommend referring to the CEE guidelines (systematic map guidelines are closest to a scoping review)).** *This protocol followed the JBI methodology for scoping reviews. We preferred to perform a scoping review (rather than a systematic map) because the aim of our study was both the exploration and the mapping of the existing evidence.  Scoping reviews aim to map the existing literature on a broad topic, identify key concepts, and determine the extent and nature of the available evidence. On the other hand, systematic maps focus on identifying and cataloguing the existing evidence on a specific topic with a primary emphasis on highlighting gaps in the evidence base.* *Among the various available methodologies for systematic evidence synthesis, we chose to follow the JBI methodology and not for example the CEE guidelines, which are specifically designed for environmental-oriented systematic synthesis. The choice of a methodology for conducting scoping reviews over others depends on various factors, including the research question, available resources, and the preferences of the researchers. Below we list the main reasons why we preferred to follow the JBI methodology: 1. Systematic Framework: JBI provides a systematic and transparent framework for conducting scoping reviews. The step-by-step approach outlined in the JBI methodology helps ensure a rigorous and structured process from defining the research question to reporting the results.2. International Collaboration: The JBI methodology is associated with an international collaboration of researchers, educators, and clinicians. This collaborative network contributes to the development and refinement of methodologies, ensuring a global perspective on evidence synthesis.3. Evidence-Based Practice: JBI is known for its commitment to evidence-based practice. The methodology emphasizes a systematic and evidence-based approach to literature synthesis, which can be appealing to researchers who prioritize a robust and rigorous review process. 4. Clear Guidelines: JBI provides clear and detailed guidelines for each step of the scoping review process. This can be particularly helpful for researchers who are new to conducting scoping reviews or those who appreciate a structured and methodical approach.5. Adaptability: The JBI methodology is designed to be adaptable to various research questions and topics. It allows for flexibility in study selection, data extraction, and synthesis, making it applicable to a wide range of disciplines and research areas. 6. Recognition and Endorsement: The JBI methodology is recognized and endorsed by the Joanna Briggs Institute, a reputable organization in the field of evidence-based healthcare. Researchers and practitioners may find comfort in using a methodology associated with a well-regarded institution.7. Education and Training: JBI offers education and training programs for researchers interested in using their methodologies. This support can be beneficial for researchers seeking guidance and expertise in conducting scoping reviews.* *Based on the above mentioned, we believe it is best to keep using the term “scoping review” as the type of evidence synthesis we performed and the” JBI methodology” throughout the text, instead of the “Systematic map” and the “CEE guidelines” respectively, that you propose.* **Comment 2. Given that this is a Scoping Review I do not understand how the authors intend to achieve objective 5 - "What are the good practices and failures acknowledged in the literature?". There is no critical appraisal of the studies included proposed and therefore it would be difficult to assess (beyond what the individual study authors suggest in each included paper) what is "good practice" (or "bad practice").** *Thank you for this important comment. As critical appraisal in scoping reviews is not mandatory, we have not planned to perform it. Thus, following your comment, we delete objective no 4 and review question no 5 from the revised manuscript (see Introduction and Review question sections).* **Comment 3. I find the structure of the protocol a bit odd - I would expect to read about the search strategy first and then read about inclusion criteria - but perhaps this is a set template.** *The structure followed for this scoping review protocol is set by the JBI methodology and the SUMARI Protocol Template for Scoping Reviews in Word format ( https://sumari.jbi.global/), which was used to guide the protocol development.* **Comment 4. There are some subheadings that appear a little redundant which might be due to a template being used - for example "Types of sources" doesn't include the actual databases (but talks about databases) and it is only when you get to the search strategy that this is mentioned.** *Again, this has to do with the JBI methodology and the SUMARI Protocol Template for Scoping Reviews that was used. According to the SUMARI protocol template guidelines Based on the JBI methodology manual (available here: https://jbi-global-wiki.refined.site/space/MANUAL), within the “Inclusion/ exclusion criteria” section you need to include a subsection entitled “Types of evidence sources”, where the following information advice is given: “For the purposes of a scoping review, the “source” of information can include any existing literature, e.g. primary research studies, systematic reviews, meta-analyses, letters, guidelines, websites, blogs, etc. Reviewers may wish to leave the source of information “open” to allow for the inclusion of any and all types of evidence. Otherwise, the reviewers may wish to impose limits on the types of sources they wish to include. This may be done on the basis of having some knowledge of the types of sources that would be most useful and appropriate for a particular topic. For example, the scoping review example on quality of life questionnaires available for pediatric patients following tonsillectomies with or without adenoidectomies for chronic infection or sleep-disordered breathing sought quantitative studies, specifically; experimental and epidemiological study designs including randomized controlled trials, non-randomized controlled trials, quasi-experimental, before and after studies, prospective and retrospective cohort studies, case-control studies, and analytical cross-sectional studies. Qualitative studies, reviews, and conference abstracts were excluded.”. Also, the methodology proposes that the databases that will be searched for eligible documents should be acknowledged in the Search strategy subsection. So as the guidelines of the JBI methodology are fully respected, we prefer not to perform any changes at the subheadings and the content of the respective subsections.* **Comment 5. There is considerable scope for streamlining the document. Another example: "Language limitations will be applied during the literature search process to align with language competence of the authors. Consequently, studies published in languages other than English, French, German, Greek, Italian, and Spanish will be excluded from the ScR." Why not: "To align with language competence of the authors only studies written in English, French, German, Greek, Italian, and Spanish will be included".** *The text was revised as suggested (see sentence of the “Types of sources” subsection of the revised manuscript).* **Comment 6. Study/source of evidence selection - if the authors plan to do forward and backward citation chasing then I recommend using citation chaser | Evidence Synthesis Hackathon (eshackathon.org)** *Thank you for this recommendation, it will be seriously considered, and if used, it will be cited in the final review paper.* **Comment 7. What is the data extraction tool? Do the authors have an example (even if not complete)? I see that the tool is linked in the data availability - but I would like to see this in the protocol explicitly.** *A data extraction tool is a structured form/template designed to systematically collect relevant information from each included study in a systematic review. The purpose of a data extraction tool is to ensure consistency in the extraction process, facilitate data synthesis, and minimize bias by clearly documenting key details from each study. There are various tools available, and researchers may choose or develop one based on their specific needs. We preferred using α Microsoft Excel spreadsheet to create our own customized data extraction form as it allows for flexibility in design, it is customizable and widely accessible. As it is described in the manuscript (see “Data extraction” subsection), this tool will be integrated into the Covidence systematic review management software, to ensure consistency and facilitate collaboration and interaction among reviewers. The data extraction tool was initially included as one of the protocol’s tables, but it was then linked to the data availability section of the manuscript following the suggestion of the editorial team during the editorial check process of the manuscript prior to its publication. If you wish to have a look at the data extraction tool you can find it available open-access here: https://doi.org/10.17605/OSF.IO/3WK5H. The link was included in the submitted manuscript (see Data availability/ Extended data subsection of the manuscript).* **Comment 8. There are some vague statements in the Data analysis section - for example "The data will be presented using a combination of graphical and tabular formats, utilizing appropriate software packages and tools. Graphical representations, such as charts, graphs, or diagrams, will be used to visually display relevant information and trends identified in the included studies. These visuals can help convey patterns, relationships, and key findings effectively.”. How will data be presented using what tools and to what aims? I know there needs to be some uncertainty here but the authors can provide more specific details here as at the moment the text is generic and uninformative.** *More details on how the data will be presented, the tools that will be used and the aim of the visualizations that will be produced and presented in the final review paper were added (see “Data analysis and presentation” section of the revised manuscript).*

# Reviewer report: Reviewer response for version 1

**DOI:** 10.21956/openreseurope.17399.r34545

**Published:** 2023-09-07

**Authors:** Jansen Smith

**Affiliations:** 1 Earth and Environmental Sciences, University of Minnesota Duluth, Duluth, MN, USA

Petza *et al. *detail a study protocol for a future Scoping Review to evaluate Other Effective area-based Conservation Measures (OECMs). OECMs represent a potentially large contribution to conserving biodiversity, especially with respect to targets related to spatial coverage and connectivity. OECMs are an intriguing and somewhat non-traditional component to conservation as they tend to be in land-use areas that are not designated for conservation but nonetheless function to conserve various aspects of biodiversity. A scoping review on OECMs is timely, as the reality of the biodiversity crisis necessitates creative and outside-the-box solutions. The assembled, multi-national and multi-lingual team has put forth a sound protocol that is well aligned with their research objectives and questions. The proposed review should provide a strong basis for integrating OECMs into ongoing and future conservation strategies, and may serve to facilitate the recognition of OECMS in Europe where none are currently recognized.

 A few additional comments for the authors to consider:

1. The main text includes German as a search language but the abstract does not. Given the inclusion of an author with a German affiliation, I assume this language should be included in the abstract. 
2. There are no data in this Study Protocol, which is logical given the article type. Still, it may be worth including a brief description of how the data collected in the Scoping Review will be shared, if at all. The "Data analysis and presentation" section mentions the presentation of data in a variety of formats, including "tabular formats" but it is not clear if this would be the raw data or summarized data. A clearer statement on the future data availability would be well aligned with this publication venue.
3. The authors may wish to provide more detail in the "Data analysis and presentation" section, in general. Whereas I appreciate that data visualization will be shaped by the data that are collected, the current description is vague to the point that it is not particularly worthwhile (e.g., "Graphical representations, such as charts, graphs, or diagrams, will be used..."). Perhaps the authors could point to some good examples from other published works that represent their intended outputs? This is a very minor issue but would provide more clarity to a future reader.
4. I greatly appreciated the "Data Extraction Tool." This provided concrete, detailed information that would make this protocol reproducible.
5. In Table 1, it seemed somewhat unnecessary to include the column "Exclusion criteria," as it was entirely empty. Will this be filled during the course of the review? It would seem that an exclusion for the Concept row was included in the text: "...except for evidence synthesis such as systematic, scoping, rapid, and narrative reviews."

This will be a large and valuable undertaking and I look forward to seeing the finished product when it is available.

Is the study design appropriate for the research question?

Yes

Is the rationale for, and objectives of, the study clearly described?

Yes

Are sufficient details of the methods provided to allow replication by others?

Yes

Are the datasets clearly presented in a useable and accessible format?

Not applicable

Reviewer Expertise:

My main area of expertise is in a subfield of conservation science called conservation paleobiology. Through research in this area, and through projects in related areas of study in biological sciences, I have conducted scoping reviews, including using similar methods to those described herein.

I confirm that I have read this submission and believe that I have an appropriate level of expertise to confirm that it is of an acceptable scientific standard.

# Response

**Published:** 2023-11-30

**Authors:** Dimitra Petza

**Affiliations:** Department of Marine Sciences, University of the Aegean, Lesvos, Mytilene, Greece

*We would like to thank the reviewer for the insightful comments. Our responses are provided in the following text, where we tried to address all comments one by one, revising also the manuscript accordingly.* **Comment 1: The main text includes German as a search language, but the abstract does not. Given the inclusion of an author with a German affiliation, I assume this language should be included in the abstract.** *The German language has been included in the abstract of the revised manuscript.* **Comment 2. There are no data in this Study Protocol, which is logical given the article type. Still, it may be worth including a brief description of how the data collected in the Scoping Review will be shared, if at all. The "Data analysis and presentation" section mentions the presentation of data in a variety of formats, including "tabular formats" but it is not clear if this would be the raw data or summarized data. A clearer statement on the future data availability would be well aligned with this publication venue.** *In fact, there is data of this Study Protocol available open-access via Open Science Framework (DOI 10.17605/OSF.IO/SEJ84), the Data Extraction Tool and the Scoping Review PRISMA Checklist. The full set of the raw data that will be collected will be also available open-access as a supplementary to the final review paper. The text was revised accordingly (see “Data Analysis and Presentation” section of the revised manuscript).* **Comment 3. The authors may wish to provide more detail in the "Data analysis and presentation" section, in general. Whereas I appreciate that data visualization will be shaped by the data that are collected, the current description is vague to the point that it is not particularly worthwhile (e.g., "Graphical representations, such as charts, graphs, or diagrams, will be used..."). Perhaps the authors could point to some good examples from other published works that represent their intended outputs? This is a very minor issue but would provide more clarity to a future reader.** *The text was revised based on this comment. More details on the types of graphs and visualizations that will be used along with some examples of the available tools that might be used for data presentation were added to the revised manuscript (see “Data Analysis and Presentation” section of the revised manuscript).* **Comment 4. I greatly appreciated the "Data Extraction Tool." This provided concrete, detailed information that would make this protocol reproducible.** *The authors wish to thank you for this comment.* **Comment 5. In Table 1, it seemed somewhat unnecessary to include the column "Exclusion criteria," as it was entirely empty. Will this be filled during the course of the review? It would seem that an exclusion for the Concept row was included in the text: "...except for evidence synthesis such as systematic, scoping, rapid, and narrative reviews."** *This exception of evidence synthesis such as systematic, scoping, rapid, and narrative reviews is indeed an exclusion criterion of this scoping review, which has been overlooked by Table 1. Thank you for this comment. This has been added in the exclusion criteria column of Table 1 in the last row” Evidence types and sources” (see Table 1 of the revised manuscript).*
